# The Relationship Between the Big Five Personality Traits and the Theory of Planned Behavior in Using Mindfulness Mobile Apps: Cross-sectional Survey

**DOI:** 10.2196/39501

**Published:** 2022-11-30

**Authors:** Sunghak Kim, Jin Young Park, Kyungmi Chung

**Affiliations:** 1 Institute of Behavioral Science in Medicine Yonsei University College of Medicine Yonsei University Health System Seoul Republic of Korea; 2 Department of Psychiatry, Yongin Severance Hospital Yonsei University College of Medicine Yonsei University Health System Yongin Republic of Korea; 3 Center for Digital Health, Yongin Severance Hospital Yonsei University College of Medicine Yonsei University Health System Yongin Republic of Korea

**Keywords:** personality traits, Theory of Planned Behavior, mindfulness, mobile apps, mental health

## Abstract

**Background:**

Mindfulness has emerged as a promising approach toward improving mental health. Interest in mindfulness mobile app services has also increased in recent years. Understanding the determinants of mindfulness behavior is essential to predict people’s utilization of mindfulness mobile apps and beneficial for developing and implementing relevant intervention strategies. Nevertheless, little has been done to determine the predictors of mindfulness behavior.

**Objective:**

This study investigates the association between the Big Five personality traits and the Theory of Planned Behavior (TPB) variables in the context of using mindfulness mobile apps to explore the potential indirect effects of conscientiousness and neuroticism on people’s behavioral intention for mindfulness, mediated by their attitude toward mindfulness, subjective norm about mindfulness, and perceived behavior control over mindfulness.

**Methods:**

The authors conducted an online, cross-sectional survey in December 2021. Structural equation modeling was conducted to evaluate the overall model fit and test possible linkages among conscientiousness, neuroticism, attitude toward mindfulness, subjective norm about mindfulness, perceived behavior control over mindfulness, and behavioral intention for mindfulness. Bootstrapping mediation analyses were also conducted to test the potential mediating effect in the model.

**Results:**

A total of 297 Korean participants’ responses (153 males and 144 females) were analyzed. The proposed model had a good fit. Conscientiousness was correlated with attitude toward mindfulness (β=.384, *P*<.001), subjective norm about mindfulness (β=.249, *P*<.001), and perceived behavior control over mindfulness (β=.443, *P*<.001). Neuroticism was not correlated with attitude toward mindfulness (β=−.072, *P*=.28), but was correlated with subjective norm about mindfulness (β=.217, *P*=.003) and perceived behavior control over mindfulness (β=−.235, *P*<.001). Attitude toward mindfulness (β=.508, *P*<.001), subjective norm about mindfulness (β=.132, *P*=.01), and perceived behavior control over mindfulness (β=.540, *P*<.001) were separately correlated with behavioral intention for mindfulness. Conscientiousness was not directly correlated with behavioral intention for mindfulness (β=−.082, *P*=.27), whereas neuroticism was directly correlated with behavioral intention for mindfulness (β=.194, *P*=.001). Conscientiousness was indirectly linked with behavioral intention for mindfulness through attitude toward mindfulness (B=0.171, 95% CI 0.103-0.251) and perceived behavior control over mindfulness (B=0.198, 95% CI 0.132-0.273) but not through subjective norm about mindfulness (B=0.023, 95% CI −0.002 to 0.060). Neuroticism was indirectly linked with behavioral intention for mindfulness via perceived behavior control over mindfulness (B=−0.138, 95% CI −0.197 to −0.088) but not via subjective norm about mindfulness (B=0.021, 95% CI −0.002 to 0.059).

**Conclusions:**

The results show that the integration of the Big Five personality traits and TPB constructs is useful in predicting the use of mindfulness mobile apps. Focusing on conscientiousness and neuroticism in developing information dissemination and implementation strategies for enhancing mindfulness behavior using mobile apps may lead to the successful promotion of mindfulness mobile apps and adherence to mindfulness techniques.

## Introduction

During the COVID-19 pandemic, the prevalence of mental health disorders, such as mood and anxiety disorders, has increased globally [1,2]. As interest in mental health promotion increases, mindfulness meditation can be introduced as an evidence-based intervention to reduce psychological distress and alleviate the psychological impact of the pandemic and long-term quarantine measures [3-5]. Reflecting the ability to pay full attention to the present moment without any judgment and not be overly reactive or overwhelmed by past or future events [6,7], mindfulness has been shown to have positive effects on mental health and psychological well-being [8-11]. As improved mental health is associated with better health behaviors, mindfulness, which reduces psychological distress, can play a key role in facilitating health behaviors [12-14]. The rapid development of information and communications technologies has led to the expansion of eHealth and mobile health (mHealth) and diverse mobile apps that contain mindfulness content. Fact.MR [15] has estimated that the market for mindfulness meditation apps is expected to reach US $180 million by 2032 with an annual compound growth rate of 8.4%. The amount of research examining the effectiveness of mindfulness mobile apps in mental health is also increasing [16-19]. Nevertheless, further research is required on the design of apps and factors that affect their use to connect this interest in mindfulness mobile apps to the development of successful mHealth intervention strategies [20-22]. Further, it is important to identify the determinants of mindfulness behavior that potentially drive the use of mindfulness mobile apps.

This paper focuses on behavioral intentions, one of the factors that enable the prediction of actual behavior. By exploring the determinants of behavioral intentions for mindfulness, this research aims to identify the factors that affect not only behavioral intentions for mindfulness but also adherence to mindfulness. To this end, the Theory of Planned Behavior (TPB), which deals with the role of behavioral intentions in actual behaviors, was adopted as the study’s core theory. The TPB is a socio-psychological model that was developed to examine the psychological processes that influence behavior. This theory claims that attitude toward behavior, subjective norm about behavior, and perceived behavior control could impact the intention to perform the behavior so as to eventually change actual behavior [23]. It defines attitude as the overall evaluations of a person engaging in a single behavior or a set of behaviors. Subjective norm refers to a person’s belief about whether others think that they should engage in a particular behavior. Perceived behavior control relates to a person’s perception of how easy or difficult it would be to perform the behavior [24]. The TPB states that engaging in a behavior depends on the relationship between these fundamental concepts and their rational processes; strong behavioral intention incurred by attitude, subjective norm, and perceived behavior control is likely to make people perform the behavior [24,25]. The TPB has been widely used to explain and predict various health behaviors [26], including smoking [27], drinking [28], and exercise [29]. In terms of mindfulness, a study of individuals with no prior experience of mindfulness meditation found that subjective norm was a predictor of people’s phone app usage time for practicing mindfulness, and that attitude and perceived behavior control were positively associated with an intention to practice mindfulness [30]. Although not focusing specifically on mindfulness behavior, another study showed that attitude and perceived behavior control predicted intention to seek mental health services [31]. As mindfulness and mental health are closely linked, it is possible to assume that the determinants of intention to seek mental health services would tend to be similar to the determinants of intention to practice mindfulness. Thus, the TPB is a useful theoretical framework for predicting mindfulness-related behavior.

There have been attempts to use the TPB to determine additional factors associated with mental health–related behaviors, thereby expanding the boundaries of the TPB and increasing the explanatory power of the theory. The Big Five personality traits (ie, openness, conscientiousness, neuroticism, extraversion, and agreeableness) [32] are representative variables. Openness describes a person’s disposition toward doing new things and intellectual activities; people with a high level of openness tend to be creative, imaginative, and curious. Conscientiousness refers to a person’s tendency to regulate themselves to perform goal-directed behaviors; people with a high level of conscientiousness are more inclined to be organized, self-disciplined, and competent. Neuroticism is related to a person’s perception of the world and overall emotional stability; people with a high level of neuroticism tend to be emotionally vulnerable, anxious, and experience a lot of stress. Extraversion describes a person’s willingness to interact with their environment; people with a high level of extraversion tend to be sociable, outgoing, and seek excitement. Agreeableness reflects the way people manage their relationships with others; people with a high level of agreeableness are more inclined to be sympathetic, cooperative, and trusting [32-34]. Previous studies have reported that these personality traits influence TPB variables and behavioral intention [35-38]. For example, affective attitudes and perceived behavioral control mediate the relationship between conscientiousness and intention for physical activity [38].

Previous mindfulness studies investigating the relationship between the Big Five personality traits and the TPB are relatively insufficient compared with studies on other health behaviors. However, considering the close correlation between the characteristics of the Big Five personality traits and mindfulness [39,40], there should be a similar close relationship between the Big Five personality traits and the TPB in the context of mindfulness behavior. For instance, conscientious people tend to be responsible and have good impulse control to achieve a goal. Mindful people are more likely to respond deliberately and carefully rather than impulsively and habitually. Thus, it is feasible to assume that conscientiousness and mindfulness have a positive relationship [39]. People with neuroticism tend to be more susceptible to stress and negative emotions and experience dramatic changes in their feelings, while mindful people are more likely to be calm and able to control their negative emotions. Therefore, it can be assumed that neuroticism and mindfulness have a negative relationship [39]. Despite the lack of evidence, we suppose that the Big Five personality traits and the TPB still have the potential to predict or change mindfulness behavior.

This paper investigates the mediation relationship in which the Big Five personality traits influence the factors of the TPB and ultimately affect behavioral intentions for mindfulness. This study reveals the mechanisms of the TPB with consideration to personality traits in the context of mindfulness behavior, thus enhancing the theoretical framework by embracing new potential variables that can predict behavioral intention. As conscientiousness and neuroticism display a stronger relationship with mindfulness than openness, extraversion, and agreeableness [39], this research focuses on the influence of conscientiousness and neuroticism on the TPB.

## Methods

### Study Design and Procedure

We conducted an online survey of adults to examine their experiences of mindfulness. Before participating in the survey, the participants were asked to read the research information and complete an electronic consent form. Only consenting participants were allowed to continue with the study. They provided sociodemographic, clinical, and individual difference information relevant to mindfulness. The questionnaire items were translated from English to Korean and modified to fit the context of the study’s topic.

### Recruitment

Participants were recruited in December 2021 using an online panel developed by a survey company, dataSpring Korea, Inc. Adults older than 19 years and who consented to participate in the survey were recruited. Based on their responses to the survey’s screening question, they were categorized into 4 groups: (1) *mindfulness mobile app users*—those who used mindfulness mobile apps within a month of either downloading an app or subscribing to a paid premium service; (2) *mindfulness mobile app churners*—those who had experience of using mindfulness mobile apps in the past but either deleted the apps or did not use them within a month of downloading them; (3) *other mindfulness behavior performers*—those who did not have any experience of using mindfulness mobile apps but had experience of practicing mindfulness behaviors through other means; and (4) *no mindfulness behaviors*—those with no experience in performing any type of mindfulness behavior.

After the survey, participants were recategorized into 4 groups: (1) people using mindfulness mobile apps and practicing other types of mindfulness behaviors; (2) people using only mindfulness mobile apps; (3) people who only practice other types of mindfulness behavior; and (4) people not practicing any type of mindfulness behavior, with a focus on current (within 1 month) mindfulness activities. Participants only practicing other types of mindfulness behaviors were excluded from the data analysis because the study focuses on the use of mindfulness mobile apps. Consequently, the responses of 297 participants were used for the data analysis. At the time of the survey, 142/297 (47.8%) participants were using mindfulness mobile apps (either in conjunction with other types of mindfulness behavior or using mindfulness mobile apps only) and 155/297 (52.2%) were not practicing any type of mindfulness behavior. Of the 142 participants using mindfulness mobile apps, 46 (32.4%) were using Mabo, 46 (32.4%) were using Kokkiri, 17 (12%) were using Calm, 13 (9.2%) were using Harumeditation, and 20 (14.1%) were using other mindfulness mobile apps.

Of the total 297 participants, 47 (15.8%) were 20-29 years old, 68 (22.9%) were 30-39 years old, 108 (36.4%) were 40-49 years old, 72 (24.2%) were 50-59 years old, and 2 (0.7%) were 60 years old or older. In addition, 153 (51.5%) participants were male and 144 (48.5%) were female; 180 (60.6%) participants were married and 102 (34.3%) were single; and 170 (57.2%) participants had no religious affiliation. More than half of the participants were university graduates (180/297, 60.6%). Regarding the participants’ employment status, 192 (64.6%) were permanently employed. More than half of the participants (188/297, 63.3%) responded that they were in the middle-income bracket. Table 1 presents more information about participants’ demographic characteristics.

**Table 1:** Demographic information of participants (N=297).

Characteristics			Participants, n (%)^a^
**Age (years)**			
	20-29	47 (15.8)	
	30-39	68 (22.9)	
	40-49	108 (36.4)	
	50-59	72 (24.2)	
	≥60	2 (0.7)	
**Gender**			
	Male	153 (51.5)	
	Female	144 (48.5)	
**Marital status**			
	Single (never married)	102 (34.3)	
	Domestic partnership/common law marriage	4 (1.3)	
	Married	180 (60.6)	
	Divorced	11 (3.7)	
**Religion**			
	None	170 (57.2)	
	Protestant	40 (13.5)	
	Catholic	36 (12.1)	
	Buddhist	49 (16.5)	
	Cheondoist	1 (0.3)	
	Won Buddhist	1 (0.3)	
**Highest level of education**			
	High school graduate	48 (16.2)	
	College graduate (2-3 years)	39 (13.1)	
	University graduate (4-6 years)	180 (60.6)	
	Master’s degree	26 (8.8)	
	Doctorate	4 (1.3)	
**Current employment status**			
	Permanently employed	192 (64.6)	
	Temporarily employed (eg, part-time workers, dispatched workers, daily workers, freelancers)	54 (18.2)	
	Not employed	49 (16.5)	
	Retired	2 (0.7)	
**Income**			
	Very low	12 (4)	
	Low	80 (26.9)	
	Middle	188 (63.3)	
	High	15 (5.1)	
	Very high	2 (0.7)	
**Current experience of mobile mindfulness apps (within 1 month)**			
	Yes	142 (47.8)	
	No	155 (52.2)	
**Experience of being diagnosed with a psychiatric disease**			
	Yes	45 (15.2)	
	No	252 (84.8)	
**Experience of being diagnosed with a medical or surgical condition**			
	Yes	143 (48.1)	
	No	154 (51.9)	
**Experience of taking medication (within the last 30 days)**			
	Yes	131 (44.1)	
	No	166 (55.9)	

^a^Percentages may not add up to 100% due to rounding.

### Measures

#### Overview

In this study, conscientiousness and neuroticism were chosen as exogenous variables and behavioral intention for mindfulness as an endogenous variable. Attitude, subjective norm, and perceived behavior control regarding mindfulness were intermediate variables.

#### Personality Factor

The Korean Big Five Inventory (BFI)-15 was used to assess respondents’ Big Five personality traits. The Korean BFI-15 was translated, abbreviated, and verified in Korean by Kim and colleagues [41] from John and Srivastava’s BFI items [42]. Along with the stem question “I see myself as someone who,” the Korean BFI-15 includes 15 items as follows: 3 items for openness, 3 items for conscientiousness, 3 items for neuroticism, 3 items for extraversion, and 3 items for agreeableness. Each item is answered on a 5-point scale (1=“strongly disagree” to 5=“strongly agree”). The responses to the conscientiousness and neuroticism items were used in the data analysis.

- Conscientiousness: Respondents were asked to indicate their conscientiousness based on 3 items: “I see myself as someone who does a thorough job,” “...does things efficiently,” and “...is a reliable worker.” These items were averaged to create a scale (mean 3.67, SD 0.77; Cronbach α=.840).
- Neuroticism: Neuroticism was measured using 3 items: “I see myself as someone who gets nervous easily,” “...is depressed, blue,” and “...worries a lot.” These items were averaged to create a scale (mean 2.79, SD 1.04; Cronbach α=.876).

#### Behavioral Factor

We modified the TPB measurement developed by Kim [43] to predict drug users’ intention to use treatment services for drug addiction to suit the context of mindfulness behavior in this study. Kim’s TPB measurement was developed and verified based on methods and evidence related to the TPB as posited by Fishbein and Ajzen [25]. The measurement in this study assesses respondents’ attitude, subjective norm, perceived behavior control, and behavioral intention regarding mindfulness.

- Attitude toward mindfulness: Five items, based on a 5-point scale (1=“strongly disagree” to 5=“strongly agree”), were modified in the context of mindfulness: “It is worthwhile to perform mindfulness,” “It is wise to perform mindfulness,” “It is practical to perform mindfulness,” “It is desirable to perform mindfulness,” and “I am positive about performing mindfulness.” The items were used to measure respondents’ attitude toward mindfulness and averaged to create a scale (mean 4.10, SD 0.63; Cronbach α=.878).
- Subjective norm about mindfulness: Four items, based on a 5-point scale (1=“strongly disagree” to 5=“strongly agree”), were modified in the context of mindfulness: “Most people who are important to me think that I need to perform mindfulness,” “Most people who are important to me would endorse me performing mindfulness,” “Most people who are important to me would support me performing mindfulness,” and “I feel pressure to perform mindfulness from people around me.” The items were used to measure respondents’ subjective norm about mindfulness and averaged to create a scale (mean 3.19, SD 0.82; Cronbach α=.770).
- Perceived behavior control about mindfulness: Three items, based on a 5-point scale (1=“strongly disagree” to 5=“strongly agree”), were modified in the context of mindfulness: “I am confident in performing mindfulness,” “It is entirely up to me to perform mindfulness,” and “I can control the situation around me to perform mindfulness.” The items were used to measure respondents’ perceived behavior control about mindfulness and averaged to create a scale (mean 3.84, SD 0.66; Cronbach α=.744).
- Behavioral intention for mindfulness: Five items, based on a 5-point scale (1=“strongly disagree” to 5=“strongly agree”), were modified in the context of mindfulness: “I intend to perform mindfulness,” “I will perform mindfulness,” “I plan to perform mindfulness,” “I want to perform mindfulness,” and “I am willing to perform mindfulness.” The items were used to measure respondents’ behavioral intention for mindfulness and averaged to create a scale (mean 3.91, SD 0.72; Cronbach α=.901).

### Statistical Analysis

Structural equation modeling (SEM) was conducted to verify the proposed research model established to predict mindfulness behavior by integrating the Big Five personality traits and the TPB. First, a confirmatory factor analysis was conducted to examine the measurement model and assess the model fit using several goodness-of-fit indices. Next, SEM was conducted to evaluate the structural model and test the study hypotheses. Lastly, bootstrapping mediation analyses were conducted to test the significance of the mediation pathways more precisely. SPSS 22.0 (IBM, Inc.), Amos 22.0 (IBM, Inc.), and PROCESS macro for SPSS 4.1 [44] software were used for the data analysis.

### Ethics Approval

This study was approved by the Institutional Review Board of Yongin Severance Hospital in Yonsei University Health System (IRB No. 9-2021-0167). We made a data collection request to an online panel research company. Only the online panel members older than 19 years had access to this study’s online survey. The members interested in the survey could thoroughly review the study explanation and voluntarily decide to participate. They could participate in the survey only if they provided informed voluntary consent. However, participants could withdraw their consent or stop participating in the study at any time according to their free will. This study was conducted through a survey; no special side effects or physical damage was expected. We received deidentified raw data from the online panel research company after the survey completion. The data were password protected, and only the research team had access.

## Results

### Scale Validation and Model Specification

A confirmatory factor analysis was conducted to verify the factor structure of the proposed model. First, after examining the factor loading of the observed variables constituting the latent variables, all the factor loading values were found to be statistically significant (*P*<.05 in all cases). The factor loading values of all the observed variables were more than 0.50, except for an observed variable in subjective norm about mindfulness (ie, the “'I feel pressure to perform mindfulness from people around me” item), which was below 0.50. Therefore, we concluded that the observed variables make up the latent variables based on an appropriate theoretical conceptualization [45,46]. In other words, content validity was established.

Next, convergent validity—the explanatory power and validity of the latent variables itself—was examined through composite/construct reliability (CR), representing the internal consistency of the observed variables, and the average variance extracted (AVE) value, representing the size of the variance that the observed variables can explain. The convergent validity was secured for all the variables because the CR exceeded 0.70 and the AVE exceeded the threshold of 0.50 [46,47].

The details of each observed and latent variable are summarized in Table 2.

After examining convergent validity, discriminant validity was examined to determine whether there was an overlap or similarity between each latent variable and whether there was differentiation. Each AVE value had to be greater than the square value of the correlation coefficient between certain variables to secure discriminant validity [47]. In this measurement model, discriminant validity was secured because the minimum value of the AVE (0.502) was greater than the largest square value of the correlation coefficient (maximum=0.696×0.696=0.484).

In terms of reliability, the Cronbach α value of each variable measurement was calculated to examine the internal consistency of the measurement tool. As the Cronbach α values of all the variables were above .70, all the measurements showed good reliability [45,48].

After confirming that the study’s measurement model met the validity and reliability requirements, an evaluation to test overall model fit was conducted. As a result, the values *χ*^2^_215_=431.1 (*P*<.001), *χ*^2^/*df*=2.005, incremental fit index (IFI)=0.942, comparative fit index (CFI)=0.941, Tucker-Lewis index (TLI)=0.930, and root mean square error of approximation (RMSEA)=0.058 met all the criteria: *χ*^2^ (*P*<.001), *χ*^2^/*df*≤3, IFI≥0.90, CFI≥0.90, TLI≥0.90, RMSEA≤0.08 [48]. Therefore, the overall model showed a good fit.

The details of the measurement model verified in this study are summarized in Table 3.

**Table 2:** Confirmatory factor analysis: items and loadings.

Construct and scale items			Factor loading^a^	SMC^b^	1 – SMC	CR^c^	AVE^d^
**Personality factor (Korean BFI^e^-15)**							
	**Conscientiousness**					0.840	0.637
		Does a thorough job.	0.780	0.608	0.392		
		Does things efficiently.	0.840	0.706	0.294		
		Is a reliable worker.	0.772	0.596	0.404		
	**Neuroticism**					0.880	0.712
		Gets nervous easily.	0.904	0.817	0.183		
		Is depressed, blue.	0.881	0.776	0.224		
		Worries a lot.	0.736	0.542	0.458		
**Behavioral factor (TPB^f^)**							
	**Attitude toward mindfulness**					0.879	0.593
		It is worthwhile to perform mindfulness.	0.768	0.590	0.410		
		It is wise to perform mindfulness.	0.803	0.645	0.355		
		It is practical to perform mindfulness.	0.718	0.516	0.484		
		It is desirable to perform mindfulness.	0.774	0.599	0.401		
		I am positive about performing mindfulness.	0.783	0.613	0.387		
	**Subjective norm about mindfulness**					0.801	0.515
		Most people who are important to me think that I need to perform mindfulness.	0.814	0.663	0.337		
		Most people who are important to me would endorse me performing mindfulness.	0.851	0.724	0.276		
		Most people who are important to me would support me performing mindfulness.	0.705	0.497	0.503		
		I feel pressure to perform mindfulness from people around me.	0.421	0.177	0.823		
	**Perceived behavior control about mindfulness**					0.749	0.502
		I am confident in performing mindfulness.	0.812	0.659	0.341		
		It is entirely up to me to perform mindfulness.	0.648	0.420	0.580		
		I can control the situation around me to perform mindfulness.	0.653	0.426	0.574		
	**Behavioral intention for mindfulness**					0.903	0.651
		I intend to perform mindfulness.	0.786	0.618	0.382		
		I will perform mindfulness.	0.861	0.741	0.259		
		I plan to perform mindfulness.	0.811	0.658	0.342		
		I want to perform mindfulness.	0.790	0.624	0.376		
		I am willing to perform mindfulness.	0.775	0.613	0.387		

^a^All factor loadings are significant at *P*<.001.

^b^SMC: squared multiple correlation.

^c^CR: composite/construct reliability.

^d^AVE: average variance extracted.

^e^BFI: Big Five Inventory.

^f^TPB: Theory of Planned Behavior.

**Table 3:** Descriptive statistics and associated measures of the measurement model.

Variables	Cronbach α	Mean (SD)	AVE^a^	1	2	3	4	5	6
1. Conscientiousness	.840	3.67 (0.77)	0.637	*0.840* ^b^	−0.370	0.359	0.132	0.494	0.294
2. Neuroticism	.876	2.79 (1.04)	0.712	0.137	*0.880*	−0.211	0.130	−0.400	−0.074
3. Attitude toward mindfulness	.878	4.10 (0.63)	0.593	0.129	0.045	*0.879*	0.345	0.666	0.695
4. Subjective norm about mindfulness	.770	3.19 (0.82)	0.515	0.017	0.017	0.119	*0.801*	0.231	0.363
5. Perceived behavior control over mindfulness	.744	3.84 (0.66)	0.502	0.244	0.160	0.444	0.053	*0.749*	0.696
6. Behavioral intention for mindfulness	.901	3.91 (0.72)	0.651	0.086	0.005	0.483	0.132	0.484	*0.903*

^a^AVE: average variance extracted.

^b^Composite reliabilities are along the diagonal and represented in italic font. Correlations are above the diagonal. Squared correlations are below the diagonal.

### Hypothesis Testing

#### SEM Analysis

Based on the verification results of the measurement model, an SEM analysis was conducted to investigate the relationship between the variables and the model fit of the structural model. The values *χ*^2^_218_=524.3 (*P*<.001), *χ*^2^/*df*=2.405, IFI=0.917, CFI=0.916, TLI=0.903, and RMSEA=0.069 satisfied all the criteria: *χ*^2^ (*P*<.001), *χ*^2^/*df*≤3, IFI≥0.90, CFI≥0.90, TLI≥0.90, RMSEA≤0.08 [48] and therefore showed a good fit. We therefore concluded that the structural model had an appropriate explanatory power to test the hypotheses predicting a relationship between variables.

#### The Direct Relationships Between Personality Traits and the TPB

With respect to personality traits, the relationships between conscientiousness and attitude toward mindfulness (β=.384, *P*<.001), conscientiousness and subjective norm about mindfulness (β=.249, *P*<.001), and conscientiousness and perceived behavior control over mindfulness (β=.443, *P*<.001) were all significant and positive. The relationship between neuroticism and attitude toward mindfulness (β=−.072, *P*=.28) was not significant. However, the relationships between neuroticism and subjective norm about mindfulness (β=.217, *P*=.003) and between neuroticism and perceived behavior control over mindfulness (β=−.235, *P*<.001) were significant. The former was positive, whereas the latter was negative.

The relationships between attitude toward mindfulness and behavioral intention for mindfulness (β=.508, *P*<.001), subjective norm about mindfulness and behavioral intention for mindfulness (β=.132, *P*=.01), and perceived behavior control over mindfulness and behavioral intention for mindfulness (β=.540, *P*<.001) were significant and positive. The relationship between conscientiousness and behavioral intention for mindfulness (β=−.082, *P*=.27) was not significant; however, the relationship between neuroticism and behavioral intention for mindfulness (β=.194, *P*=.001) was significant and positive.

Table 4 summarizes all the predictable direct effect paths of this structural model.

**Figure 1:**
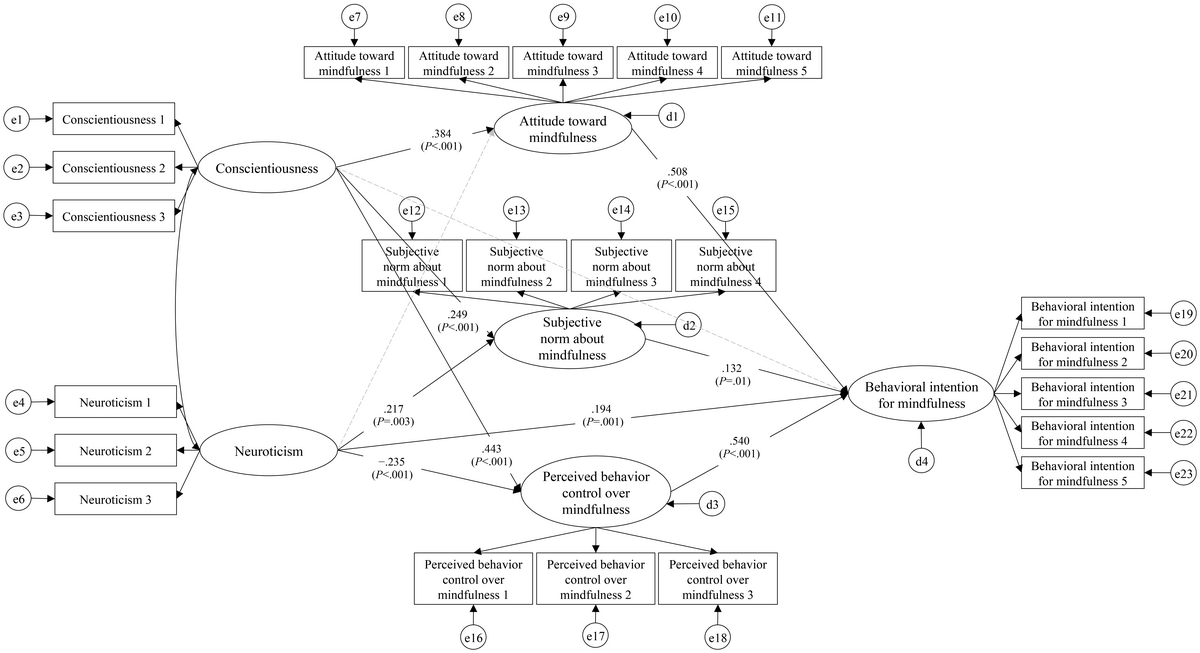
The path diagram and coefficients of the research model. e1-e23 are error terms for observed variables (ie, measurement errors); d1-d4 are disturbance terms for endogeneous variables (ie, residual errors).

The results of verifying the research model are shown in Figure 1. It offers an understanding of possible mediating mechanisms of the model.

**Table 4:** The predictable direct effect paths of the structural model.

Direct path	Coefficients (β)	*P* value	Decision
Conscientiousness → Attitude toward mindfulness	.384	<.001	Supported
Conscientiousness → Subjective norm about mindfulness	.249	<.001	Supported
Conscientiousness → Perceived behavior control over mindfulness	.443	<.001	Supported
Neuroticism → Attitude toward mindfulness	−.072	.28	Not supported
Neuroticism → Subjective norm about mindfulness	.217	.003	Supported
Neuroticism → Perceived behavior control over mindfulness	−.235	<.001	Supported
Attitude toward mindfulness → Behavioral intention for mindfulness	.508	<.001	Supported
Subjective norm about mindfulness → Behavioral intention for mindfulness	.132	.01	Supported
Perceived behavior control over mindfulness → Behavioral intention for mindfulness	.540	<.001	Supported
Conscientiousness → Behavioral intention for mindfulness	−.082	.27	Not supported
Neuroticism → Behavioral intention for mindfulness	.194	.001	Supported

#### The Indirect Relationships Between Personality Traits and the TPB

Next, the indirect effect path hypotheses were tested. Mediation analyses were conducted using the PROCESS macro model 4 (set to 5000 bootstrapped samples) [44]. In the PROCESS macro, the indirect effect is statistically significant when the CI does not include 0. Considering the paths of the model, the indirect effect path of “neuroticism → attitude toward mindfulness → behavioral intention for mindfulness” was not tested.

The results showed that conscientiousness had a potential indirect effect on behavioral intention for mindfulness mediated through attitude toward mindfulness (B=0.171, 95% CI 0.103-0.251) and perceived behavior control over mindfulness (B=0.198, 95% CI 0.132-0.273). Last, neuroticism had a potential indirect effect on behavioral intention for mindfulness mediated through perceived behavior control over mindfulness (B=−0.138, 95% CI −0.197 to −0.088). The relationships between conscientiousness and behavioral intention for mindfulness mediated by subjective norm about mindfulness (B=0.023, 95% CI −0.002 to 0.060) and between neuroticism and behavioral intention for mindfulness mediated by subjective norm about mindfulness (B=0.021, 95% CI −0.002 to 0.059) were not significant. As neuroticism was also directly associated with behavioral intention for mindfulness, neuroticism’s potential indirect effect was considered a partial mediation path. Conscientiousness’s potential indirect effects were considered full mediation paths.

Table 5 summarizes all the predictable indirect effect paths of this structural model.

**Table 5:** The predictable indirect effect paths of the structural model.

Indirect path	Coefficients (B)	Boot SE	Boot LLCI^a^	Boot ULCI^b^	Decision
Conscientiousness → Attitude toward mindfulness → Behavioral intention for mindfulness	0.171	0.037	0.103	0.251	Supported
Conscientiousness → Subjective norm about mindfulness → Behavioral intention for mindfulness	0.023	0.015	−0.002	0.060	Not supported
Conscientiousness → Perceived behavior control over mindfulness → Behavioral intention for mindfulness	0.198	0.036	0.132	0.273	Supported
Neuroticism → Subjective norm about mindfulness → Behavioral intention for mindfulness	0.021	0.015	−0.002	0.059	Not supported
Neuroticism → Perceived behavior control over mindfulness → Behavioral intention for mindfulness	−0.138	0.027	−0.197	−0.088	Supported

^a^LLCI: lower level of confidence interval.

^b^ULCI: upper level of confidence interval.

## Discussion

### Principal Findings

This research theorized and examined the indirect effect of the Big Five personality traits on behavioral intention for mindfulness based on the TPB. The study’s main finding was that conscientiousness and neuroticism could potentially impact behavioral intention for mindfulness through attitude toward mindfulness and perceived behavior control over mindfulness. The results showed that among the Big Five personality traits, only conscientiousness and neuroticism were determinants influencing people’s mindfulness behavior.

This study found no direct association between conscientiousness and behavioral intention for mindfulness; however, conscientiousness was positively associated with behavioral intention for mindfulness, via attitude toward mindfulness and perceived behavior control over mindfulness. This finding indicates that the higher the level of conscientiousness, the more positive the attitude toward mindfulness, and the stronger the perceived behavior control over mindfulness. In addition, attitude toward mindfulness and perceived behavior control over mindfulness were positively associated with behavioral intention for mindfulness, indicating that the more positive the attitude toward mindfulness and higher perceived behavior control over mindfulness, the stronger the behavioral intention for mindfulness. A strong sense of responsibility, good discipline, and effective self-regulation are therefore closely related to the characteristics of high conscientiousness [34]. People with a high level of conscientiousness are more likely to think positively about mindfulness and perceive that they are confident in performing mindfulness activities. Consequently, conscientiousness increases the likelihood of inducing behavioral intention for mindfulness.

Most importantly, neuroticism was negatively associated with behavioral intention for mindfulness via perceived behavior control over mindfulness. Inconsistent with previous studies [39,40], neuroticism was directly and positively associated with behavioral intention for mindfulness. Unlike conscientiousness, neuroticism showed a negative association with perceived behavior control over mindfulness. In other words, the higher the level of neuroticism, the weaker the perceived behavior control over mindfulness. In addition, perceived behavior control over mindfulness was positively associated with behavioral intention for mindfulness. Thus, the lower the level of perceived behavior control over mindfulness, the weaker the behavioral intention for mindfulness. As vulnerability to negative emotions is one of the characteristics of high levels of neuroticism [34], individuals with high neuroticism are less likely to perceive that they are confident in performing mindfulness. Hence, neuroticism may play a key role in reducing behavioral intention for mindfulness.

The results of this study suggest that people with high levels of neuroticism are more likely to have strong behavioral intentions for mindfulness regardless of perceived behavior control over mindfulness. One explanation is that people with high neuroticism are more likely to have poor mental health [49] but may seek treatment rather than avoidance under certain conditions. Those who perceived a high need for treatment for depression or social support are also more likely to seek professional help for depression [50]. Their strong motivation for mental health recovery may lead to stronger behavioral intention for mindfulness. In this regard, more studies are needed to verify this possible explanation and find a condition that may in turn produce results that are inconsistent with the findings of previous studies.

### Implications

In the context of mindfulness, this study suggests an integrated model to explain the relationship between personality and behavior by combining the Big Five personality traits and the TPB. The results show that personality factors (ie, conscientiousness and neuroticism) and behavioral factors (ie, attitude toward mindfulness, subjective norm about mindfulness, perceived behavior control over mindfulness, and behavioral intention for mindfulness) are closely associated with changes in mindfulness behavior.

This study shows that the relationships or patterns between the Big Five personality traits and TPB variables in the context of mindfulness differ depending on personality traits. Attitude toward mindfulness and perceived behavior control over mindfulness mediate the association between conscientiousness and behavioral intention for mindfulness. However, only perceived behavior control over mindfulness mediates the association between neuroticism and behavioral intention for mindfulness. Furthermore, 3 other Big Five personality traits (ie, openness, extraversion, and agreeableness) are known to be less associated with mindfulness [39]. We posit that such inconsistencies might stem from the different characteristics of the Big Five personality traits. For example, the characteristics of high openness and liking new and diverse experiences are quite far removed from those attributes of mindfulness that require relaxation and peace. The conflict between personality traits and mindfulness characteristics may impact their associations. Examining the underlying mechanism of the effect of personality traits’ different characteristics on mindfulness behavior could require a more advanced theoretical model with higher explanatory power on mindfulness behavior. Scrutinizing the multifaceted nature of mindfulness [51,52] may also provide a useful lens to interpret the relationship between personality traits and mindfulness. Future studies could examine the reasons why different relationships exist between personality traits and mindfulness behavior.

Our findings could also help to establish successful persuasion strategies that can encourage mindfulness behavior contingent on each individual’s personality. If health professionals are able to identify a conscientious person, they could provide an intervention that may improve their attitude toward mindfulness and perceived behavior control over mindfulness to induce behavioral intentions for mindfulness and thereby promote mindfulness behavior. If health professionals are able to identify a person with high neuroticism, they could concentrate on improving the person’s perceived behavior control over mindfulness and provide appropriate intervention effectively.

When considering mindfulness mobile apps as health intervention tools, applying immersive technology in developing mindfulness mobile apps to provide users with virtual experiences of mindfulness could be useful to improve users’ perceived behavior control over mindfulness [53]. Embedding information on personality traits when designing the content of mindfulness mobile apps could be helpful in allowing users to perceive the content as personally relevant. As personalized health programs are known to have more persuasive effects than regular health messaging [54], tailoring a health intervention based on individual characteristics may improve mindfulness behavior. If a person with high neuroticism uses a mindfulness mobile app service to practice mindfulness behavior virtually and receives mindfulness tips for people with high neuroticism, the user may be more successful in overcoming the characteristics of neuroticism and improve their perceived behavior control over mindfulness. Taken together, scholars and health practitioners need to consider the indirect effects of personality traits to influence change or adherence to mindfulness behavior.

In this study, we only explored the potential of personality and behavioral factors to impact mindfulness behavior likely to drive the use of mindfulness mobile apps. We acknowledge that demonstrating other factors impacting mHealth adoption, not limited to personality traits, is important to build on the growing body of research examining mindfulness mobile apps usage. In their systematic review, Jacob and colleagues [55] introduced social and personal factors, technical and material factors, and health-related factors affecting mHealth adoption. Demographic factors (eg, age, gender, education, ethnicity, and socioeconomic factors), personal characteristics (eg, attitudes, motivation, and psychological factors), and cultural and social elements (eg, social influence, language, and culture) were representative social and personal factors. Usefulness (eg, perceived benefit, communication, and self-management), ease of use, technical factors (eg, access to technology, training, and tech support), monetary factors, data related (eg, privacy, credibility, and relevance), and user experience (eg, usability and personalization) composed technical and material factors. Disease or health condition, care team’s role, health consciousness and literacy, relation to other therapies, health behavior, and insurance status were labeled as health-related factors [55]. Jacob and colleagues [55] emphasized taking a more holistic view of these factors and developing more patient-centered approaches (eg, fit into patient journey, inclusive design, and patient education) to facilitate mHealth adoption. They also highlighted that these factors are not mutually exclusive and showed mixed results on mHealth adoption depending on the context. It means that focusing on 1 or only a few factors alone on mHealth adoption and implementation is not likely to achieve success.

The complexity of the factors affecting mHealth adoption that Jacob and colleagues [55] stressed provides an implication to this study. The different potentials of personality and behavioral factors to impact mindfulness behavior and the use of mindfulness mobile apps can be explained by not only the characteristics of personality traits themselves but also other factors and contexts beyond personality traits. For instance, even people with high conscientiousness may only perform mindfulness behavior and use mindfulness mobile apps if they have strong motivation. They may not use mindfulness mobile apps if they feel such apps are uncomfortable to use or cannot protect personal data well. By contrast, people with low conscientiousness may use mindfulness mobile apps if they were educated about the importance of mindfulness and how to use mHealth services. This complexity may result in different and inconsistent outcomes of the associations between personality traits and mindfulness behavior. More comprehensive approaches to knowing the mediating and moderating roles of various factors are crucial to predict the use of mindfulness mobile apps. Future research is required to discover successful strategies for using the characteristics of the factors affecting mHealth adoption and their interconnectivity to facilitate the use of mindfulness mobile apps.

### Limitations

The study has a few limitations. First, this research only looked at the Big Five personality traits and TPB variables and the mediating relationships among them. The study did not consider other possible variables that could impact mediations or other possible relationships among the Big Five personality traits and TPB variables. If this deficiency can be addressed and the proposed model can be expanded through follow-up studies, our understanding of the relationship between personality traits and behavior in the context of mindfulness will be improved. Second, although this study examined mediations supported by theoretical discussions and previous evidence, a careful interpretation of the causal assumptions is merited because this study used cross-sectional data. Future studies using experimental research methods are needed to verify causal relationships. Third, considering the development of this research’s proposed model, future studies should be conducted with other types of health behaviors to refine the model or increase the model’s degree of generalizability. Fourth, this study’s participants recruited through an online panel were already likely to favor digital technology use itself. Therefore, it is encouraged to adopt a more randomized participant selection method to deal with this inherent bias and recruit participants regardless of technology preference or knowledge. Last, the data were self-reported and could be strengthened by physiological response measures.

### Conclusions

The COVID-19 pandemic has highlighted the importance of mental health and mindfulness and various types of eHealth and mHealth mindfulness services have appeared [56]. The question is how to promote mindfulness behavior and encourage people to adhere to mindfulness behavior. Identifying the determinants of mindfulness behavior is the first to answer this question. This paper focused on personality traits and verified that the combination of the Big Five personality traits and the TPB provides a useful theoretical framework for predicting mindfulness behavior. The research results provide a foundation to develop an advanced model that is able to illustrate the relationships between personality and behavioral factors by adding other potential variables and moderation paths in the context of mindfulness. The findings also underscore the potential of integrating other personality and behavior change theories to build a new model to explain mindfulness behavior. Furthermore, this research provides evidence to extend the research field and explore how diverse characteristics of personality traits affect mindfulness behavior. The role of personality may well explain mindfulness behavior more than people realize.

## Abbreviations

AVE: average variance extracted
BFI: Big Five Inventory
CFI: comparative fit index
CR: composite/construct reliability
IFI: incremental fit index
LLCI: lower level of confidence interval
mHealth: mobile health
RMSEA: root mean square error of approximation
SEM: structural equation modeling
SMC: squared multiple correlation
TLI: Tucker-Lewis index
TPB: Theory of Planned Behavior
ULCI: upper level of confidence interval

## References

[1] COVID-19 Mental Disorders Collaborators (2021). Global prevalence and burden of depressive and anxiety disorders in 204 countries and territories in 2020 due to the COVID-19 pandemic. Lancet.

[2] World Health Organization (WHO) (2022). COVID-19 pandemic triggers 25% increase in prevalence of anxiety and depression worldwide. WHO.

[3] Antonova E, Schlosser K, Pandey R, Kumari V (2021). Coping With COVID-19: Mindfulness-Based Approaches for Mitigating Mental Health Crisis. Front Psychiatry.

[4] Zhu JL, Schülke Rasmus, Vatansever D, Xi D, Yan J, Zhao H, Xie X, Feng J, Chen MY, Sahakian BJ, Wang S (2021). Mindfulness practice for protecting mental health during the COVID-19 pandemic. Transl Psychiatry.

[5] Farris SR, Grazzi L, Holley M, Dorsett A, Xing K, Pierce CR, Estave PM, O'Connell Nathaniel, Wells RE (2021). Online Mindfulness May Target Psychological Distress and Mental Health during COVID-19. Glob Adv Health Med.

[6] Mindful staff (2017). Jon Kabat-Zinn: Defining Mindfulness. Mindful.

[7] Mindful staff (2020). What is Mindfulness?. Mindful.

[8] Creswell JD (2017). Mindfulness Interventions. Annu Rev Psychol.

[9] Evans S, Ferrando S, Findler M, Stowell C, Smart C, Haglin D (2008). Mindfulness-based cognitive therapy for generalized anxiety disorder. J Anxiety Disord.

[10] Hofmann SG, Gómez Angelina F (2017). Mindfulness-Based Interventions for Anxiety and Depression. Psychiatr Clin North Am.

[11] Kabat-Zinn J, Massion AO, Kristeller J, Peterson LG, Fletcher KE, Pbert L, Lenderking WR, Santorelli SF (1992). Effectiveness of a meditation-based stress reduction program in the treatment of anxiety disorders. Am J Psychiatry.

[12] Kushlev K, Drummond DM, Diener E (2020). Subjective Well-Being and Health Behaviors in 2.5 Million Americans. Appl Psychol Health Well Being.

[13] Walsh JL, Senn TE, Carey MP (2013). Longitudinal associations between health behaviors and mental health in low-income adults. Transl Behav Med.

[14] Cohen S, Schwartz JE, Bromet EJ, Parkinson DK (1991). Mental health, stress, and poor health behaviors in two community samples. Preventive Medicine.

[15] (2022). Mindfulness Meditation Apps Market. Fact.MR.

[16] Bostock S, Crosswell AD, Prather AA, Steptoe A (2019). Mindfulness on-the-go: Effects of a mindfulness meditation app on work stress and well-being. J Occup Health Psychol.

[17] Huberty J, Green J, Glissmann C, Larkey L, Puzia M, Lee C (2019). Efficacy of the Mindfulness Meditation Mobile App "Calm" to Reduce Stress Among College Students: Randomized Controlled Trial. JMIR Mhealth Uhealth.

[18] Lyzwinski LN, Caffery L, Bambling M, Edirippulige S (2019). The Mindfulness App Trial for Weight, Weight-Related Behaviors, and Stress in University Students: Randomized Controlled Trial. JMIR Mhealth Uhealth.

[19] Taylor G, Bylund CL, Kastrinos A, Alpert JM, Puig A, Krajewski JMT, Sharma B, Fisher CL (2022). Practicing Mindfulness through mHealth Applications: Emerging Adults' Health-Enhancing and Inhibiting Experiences. Int J Environ Res Public Health.

[20] Mani M, Kavanagh DJ, Hides L, Stoyanov SR (2015). Review and Evaluation of Mindfulness-Based iPhone Apps. JMIR Mhealth Uhealth.

[21] Huberty J, Green J, Puzia M, Stecher C (2021). Evaluation of Mood Check-in Feature for Participation in Meditation Mobile App Users: Retrospective Longitudinal Analysis. JMIR Mhealth Uhealth.

[22] Plaza I, Demarzo MMP, Herrera-Mercadal P, García-Campayo Javier (2013). Mindfulness-based mobile applications: literature review and analysis of current features. JMIR Mhealth Uhealth.

[23] Ajzen I (1991). The theory of planned behavior. Organizational Behavior and Human Decision Processes.

[24] Montaño DE, Kasprzyk D, Glanz K, Rimer BK, Viswanath K (2015). Theory of Reasoned Action, Theory of Planned Behavior, and the Integrated Behavioral Model. Health Behavior: Theory, Research, and Practice.

[25] Fishbein M, Ajzen I (2009). Predicting and Changing Behavior: The Reasoned Action Approach.

[26] Godin G, Kok G (1996). The theory of planned behavior: a review of its applications to health-related behaviors. Am J Health Promot.

[27] Godin G, Valois P, Lepage L, Desharnais R (1992). Predictors of smoking behaviour: an application of Ajzen's theory of planned behaviour. Br J Addict.

[28] Collins SE, Witkiewitz K, Larimer ME (2011). The theory of planned behavior as a predictor of growth in risky college drinking. J Stud Alcohol Drugs.

[29] Shahar S, Ahmad MH, Mohd Fahmi Teng NI, Mohd Sakian NI, Omar B, Abd Manaf Z (2014). Applying theory of planned behavior to predict exercise maintenance in sarcopenic elderly. CIA.

[30] Crandall A, Cheung A, Young A, Hooper AP (2019). Theory-Based Predictors of Mindfulness Meditation Mobile App Usage: A Survey and Cohort Study. JMIR Mhealth Uhealth.

[31] Bohon LM, Cotter KA, Kravitz RL, Cello PC, Fernandez Y Garcia Erik (2016). The Theory of Planned Behavior as it predicts potential intention to seek mental health services for depression among college students. J Am Coll Health.

[32] John OP, Naumann LP, Soto CJ, John OP, Robins RW, Pervin LA (2010). Paradigm shift to the integrative big-five trait taxonomy: history, measurement, and conceptual issues. Handbook of Personality: Theory and Research.

[33] Cherry K (2022). What Are the Big 5 Personality Traits?: Openness, Conscientiousness, Extraversion, Agreeableness, and Neuroticism. Verywell Mind.

[34] Lim A (2020). The big five personality traits. Simply Psychology.

[35] Davies CA, Mummery WK, Steele RM (2010). The relationship between personality, theory of planned behaviour and physical activity in individuals with type II diabetes. Br J Sports Med.

[36] Conner M, Abraham C (2016). Conscientiousness and the Theory of Planned Behavior: Toward a more Complete Model of the Antecedents of Intentions and Behavior. Pers Soc Psychol Bull.

[37] Courneya KS, Bobick TM, Schinke RJ (2010). Does the Theory of Planned Behavior Mediate the Relation Between Personality and Exercise Behavior?. Basic and Applied Social Psychology.

[38] McEachan RR, Sutton S, Myers L (2010). Mediation of personality influences on physical activity within the theory of planned behaviour. J Health Psychol.

[39] Giluk TL (2009). Mindfulness, Big Five personality, and affect: A meta-analysis. Personality and Individual Differences.

[40] Hanley AW, Garland EL (2017). The mindful personality: A meta-analysis from a cybernetic perspective. Mindfulness (N Y).

[41] Kim JH, Kim BH, Ha MS (2011). Validation of a Korean version of the Big Five inventory. Journal of Human Understanding and Counseling.

[42] John OP, Srivastava S, Pervin LA, John OP (1999). The Big Five trait taxonomy: history, measurement and theoretical perspectives. Handbook of Personality: Theory and Research.

[43] Kim N (2018). Development of a Measurement to Predict Drug Users’ Intent to Use Treatment Services : Applying the Theory of Planned Behavior. SR.

[44] Hayes AF (2022). Introduction to Mediation, Moderation, and Conditional Process Analysis: A Regression-Based Approach.

[45] Hair JF, Black WC, Babin BJ, Anderson RE (2009). Multivariate Data Analysis.

[46] Arifin WN, Yusoff MSB (2016). Confirmatory Factor Analysis of the Universiti Sains Malaysia Emotional Quotient Inventory Among Medical Students in Malaysia. SAGE Open.

[47] Fornell C, Larcker DF (2018). Evaluating Structural Equation Models with Unobservable Variables and Measurement Error. Journal of Marketing Research.

[48] Nguyen DM, Chiu YH, Le HD (2021). Determinants of Continuance Intention towards Banks’ Chatbot Services in Vietnam: A Necessity for Sustainable Development. Sustainability.

[49] Drake MM, Morris DM, Davis TJ (2017). Neuroticism's susceptibility to distress: Moderated with mindfulness. Personality and Individual Differences.

[50] Shumet S, Azale T, Ayano G, Abebaw D, Amare T, Getnet W (2019). Intention to seek help for depression and associated factors among residents of Aykel town, Northwest Ethiopia: cross-sectional study. Int J Ment Health Syst.

[51] Bishop SR (2004). Mindfulness: A Proposed Operational Definition. Clinical Psychology: Science and Practice.

[52] Spinhoven P, Huijbers MJ, Zheng Y, Ormel J, Speckens AE (2017). Mindfulness facets and Big Five personality facets in persons with recurrent depression in remission. Personality and Individual Differences.

[53] Wienrich C, Döllinger N, Hein R (2021). Behavioral Framework of Immersive Technologies (BehaveFIT): How and Why Virtual Reality can Support Behavioral Change Processes. Front. Virtual Real.

[54] Rimer BK, Kreuter MW (2006). Advancing Tailored Health Communication: A Persuasion and Message Effects Perspective. J Communication.

[55] Jacob C, Sezgin E, Sanchez-Vazquez A, Ivory C (2022). Sociotechnical Factors Affecting Patients' Adoption of Mobile Health Tools: Systematic Literature Review and Narrative Synthesis. JMIR Mhealth Uhealth.

[56] Misitzis A (2020). Increased Interest for Mindfulness Online. Int J Yoga.

